# Homœopathy in Pain

**Published:** 1890-05

**Authors:** J. K. Mendenhall

**Affiliations:** Saratoga, N. Y.


### HOMŒOPATHY IN PAIN.

Rev. J. K. Mendenhall, Saratoga, N. Y.

The charge is often made against Homœopathy that, while it answers a good purpose ordinarily, in cases of great pain it is either powerless or of small value. In an experience, covering more than thirty years, I have found exactly the reverse of this to be true, and propose to give a few cases out of many in corroboration of this assertion.

G. D., male, age fifty-five, subject for many years to severe sciatica, which came with unfailing regularity every week. The attack lasted thirty-six hours, reaching the height of severity in about fifteen hours, continuing in its intensity six or more hours, and then gradually diminishing till the final cessation of pain. Morphine alone gave him relief; this it did promptly, but its subsequent effects were so bad that he had come to dread them almost as much as the pain.

I saw him one day when the pain was approaching its height, the time he always took the Morphine; he was about to use it then. I begged him not to do so, asked a few questions and found Arsenicum well indicated; gave one dose (Jehnichen 40m) at five P. M. Inside of two hours there was a marked relief; he slept well that night, and next morning came before I was out of bed to thank me for saving him from the horrible pain and the baleful effect of Morphine.

At the end of a week premonitory symptoms of another attack made their appearance, but a single dose of the same remedy averted it; and further, during the remainder of my stay in that place, more than a month, there was no return.

It is worthy of note that in this case $Ars.^6$ had been used during the previous winter with no results.

H. T., male, age about fifty, subject for more than fifteen years to facial neuralgia, which came weekly, first on one side and the next week on the other. While the entire side of the face was involved the centre of pain was always in the eye.

The history of each attack was almost exactly like the preceding case. When the pain was at its height it produced what the patient himself called hysteria; he laughed and cried by turns.

This case had been treated by the best allopaths of Philadelphia and Baltimore, but with no results, for the gentleman refused to take Morphine. I saw him one day after he had been suffering some twelve hours, the "hysteria" was coming, the pains in the eyes were as if "*red-hot needles were thrust into it.*" At twelve o'clock, noon, one dose Ars. (Jehnichen 40m); by one P. M. there was an amelioration. At four P. M. on my return to his house the patient was dressed, absolutely free from pain, ready for a theological discussion, of which he was very fond, full of gratitude for the cure, and equally full of wonder at it. When he sent for me he was careful also to send word that he expected no results, for he had no confidence in Homœopathy.

Mrs. R. H., age about sixty, had severe neuralgia in back of neck. She had been under the care of one of the most distinguished neurologists (allopathic) in the United States for more than a month without even the very slightest benefit. As a last resort he gave her a bottle of Collis Brown's Chlorodine, with the remark that "possibly it might do her good."

One day she appealed to me, saying that she did not believe in Homœopathy, but was willing to try it. The symptoms seemed to indicate Ars., which was given, but without result. Next day Bell. seemed to be the remedy, but it did no good. The third morning I found her with tears running down her cheeks, *filled with despair*, sobbing and exclaiming that, "*Nothing could be done,*" and apparently on the verge of *madness.* I confidently gave her Veratrum-alb., and in six hours the cure was complete and permanent.

